# Evaluation of the effects on SpO2 of N95 mask (FFP2) on dental health care providers: a cross-sectional observational study

**DOI:** 10.1186/s12913-022-07648-5

**Published:** 2022-02-24

**Authors:** Sabina Saccomanno, Rebecca Jewel Manenti, Silvia Giancaspro, Licia Coceani Paskay, Christine Sofiane Katzenmaier, Rodolfo Francesco Mastrapasqua, Vincenzo Quinzi

**Affiliations:** 1 Department of Health, Life and Environmental Science, University of L’Aquila, Piazza Salvatore Tommasi, 67100 L’Aquila, Italy; 2 Academy of Orofacial Myofunctional Therapy (AOMT), 910 Via De La Paz, Ste.106, Pacific Palisades, CA USA; 3 Speech-Language Pathologist, Singing Voice Specialist & Myofunctional Therapist in Culver City, Culver City, CA USA; 4 ENT Department, Rivoli Hospital, ASLTO3, Rivoli, Italy

**Keywords:** FFP2 mask, N95 mask: hypoxemia, Dental care providers, Pulse oximetry, Pulse oximeter, SpO^2^, Communication, COVID-19

## Abstract

**Objective:**

The purpose of this cross-sectional observational study was to evaluate the effects of SpO2 in a sample of dental health care providers who wear a N95 mask or Filtering Face Piece (FFP2) for four consecutive hours, measured by a pulse oximeter before donning the mask and again after four hours of work and to offer some strategies to minimize discomfort and improve communication with their patients while wearing the mask.

**Materials and methods:**

A 17-item questionnaire was sent via Google Drive to various practitioners in Italy and the USA. A sample of 162 questionnaires were returned from dentists, orthodontists, dental hygienists and dental assistants who committed to wearing a FFP2 for 4 consecutive hours during a work day and then measuring the oxygen saturation by way of a pulse oximeter before and after the 4 working hours. The final analysis was performer on 147 viable questionnaires returned. The sample was composed of 62 males and 85 females with an average age of 42.9 ± 12.0 years.

**Results:**

For the entire sample population, the baseline saturation was 98.6 ± 1.2 and, after four hours of mask wearing, there was a significant decrease in oxygen saturation to 97.0 ± 2.9 (*p* < 0.01). No statistical differences in SpO^2^ were found across specialties or across types of procedures performed during the 4 h. Heart rates were not significantly different before and after the 4 h in all categories. The 3 most frequent reported complaints were: fatigue (64%), headache (36%) and external ear pain (31%). The most common additional personal protective equipment (PPE) was a mask shield (78%) and those who wore the mask continuously reported more communication difficulty with patients, compared with those who took the mask off more often, in fact, 64% of the subjects reported that using the mask influenced their communication with their patients. Based on the results of the questionnaire, a list of breathing and vocal folds health strategies was devised and proposed, along with strategies to augment communication with patients.

**Conclusions:**

This study highlights a significant decrease in oxygen saturation after only 4 h of work (except for smokers) while wearing a FFP2, and confirms the widespread symptoms of fatigue, headache and pain behind the ears that dental professionals experience. But it also highlighted how mask wearing impaired communication with patients and wearing additional masks and a facial shield may add to those communications difficulties. This aspect and the need for better communication can lead the operators to remove the mask to improve breathing and communication, thus putting themselves at a risk of infection. Of all the aspects explored in this study, the most interesting was indeed the impact on fatigue and communication and the strategies proposed in this article can easily be implemented to reduce headache and fatigue by improving breathing efficiency and by aiding communication while donning a mask by improving voice quality and by using augmentative communication tools.

## Introduction

During the COVID-19 pandemic, dental offices were required to use, for all the procedures associated with aerosol production, personal safety equipment consisting of a Filtering Face Piece or FFP2/FFP3 (N95) respiratory mask, gloves, safety glasses and a waterproof overall [1]. There are different types of protection based on their purpose, such as FFP1, FFP2 and FFP3 masks.

FFP1 masks refers to the least filtering level of the three masks, with an aerosol filtration of at least 80% for 0.3 µm particles, and they are mainly used as environmental dust masks. Surgical masks FFP1 are disposable barriers that only protect from splashes and droplets of biological liquids, but not from suspended infectious agents (aerosols). The use of these masks, therefore, generally does not guarantee protection against a virus.

FFP2 masks have a minimum of 94% filtration percentage while FFP3 masks are the most filtering mask of the FFP group. With a minimum filtration percentage of 99%, they protect against very fine particles such as asbestos. The recommended masks for doctors and people in contact with COVID-19 patients are either FFP2 or FFP3 [2].

Dental health care providers wearing FFP2 masks reported frequently experiencing some physical discomfort, fatigue, and possibly even a worsening of their performance. As it is well known that heat and moisture get trapped beneath masks, it seems reasonable that some of the exhaled CO^2^ may also be trapped beneath them, inducing a decrease in blood oxygenation [3]. Thanks to pulse oximetry it is possible to detect a decrease in the percentage of oxygen saturation (SpO^2^), which normally ranges between 100% and 95% with a clinically acceptable accuracy of +/- 2% [4, 5]. When the SpO^2^ is too low (below 95%) it becomes hypoxemia, a sign of a problem linked to breathing or circulation, and may result in various symptoms, such as shortness of breath or a blue tinge of the skin, lips and mucosae. Pulse oximetry is used universally to measure arterial oxygen saturation as it is noninvasive (a clip or tape on a finger), easy to operate, and suitable for different patient populations. Pulse oximeters have some limits, which may be related to erroneous readings. Because of the sigmoid shape of the oxyhemoglobin dissociation curve, oximetry may not be able to detect hypoxemia in patients with high arterial oxygen tension (PaO^2^) levels [4]. Factors adversely affecting the accuracy of pulse oximeter output include: transducer movement, peripheral vasoconstriction, a non-pulsating vascular bed, hypotension, anemia, changes in systemic vascular resistance, hypothermia, the presence of intravascular dyes, and nail polish [6]. *Normal blood O*^*2*^* saturation* is defined as a fractional saturation of 90 to 97.5%, which corresponds to an arterial oxygen partial pressure of 13.3 to 13.7 kPa, if there are no other hemoglobin species, apart from oxy- and reduced hemoglobin.

The purpose of this cross-sectional observational study was to evaluate the effects of SpO^2^ in a sample of dental health care providers who wear a N95 mask (FFP2) for four consecutive hours, measured by a pulse oximeter before donning the mask and again after four hours of work. In addition to a reduction of SpO^2^ and its consequences, donning a mask under the specific conditions of this viral pandemic creates a physical barrier to communication with patients, especially for those who are hard of hearing. The strain applied to the vocal cords to augment communication contributes to additional symptoms such as a feeling of a dry mouth and throat, an increased feeling of fatigue and a sore throat. Because these symptoms may impact the well-being of the professionals and reduce productivity, the questionnaire sent to dental professionals tried to address communication issues as well. Therefore, a consultation was sought with a speech pathologist with expertise in voice pathologies and therapy to address this aspect of wearing these particular masks under these stringent requirements, which do not easily and safely allow the removal of the mask to improve communication with the patient, without going through an elaborate procedure about doffing (taking off) and donning (putting on) the masks.

## Materials and methods

### Guidelines

The authors devised and refined the survey’s questions keeping in mind the purpose of this study. It was decided to disseminate the questionnaire as follows: 1) directly to known professionals to share with colleagues; 2) to university dental departments, to be further disseminated among their affiliated professionals, and 3) a link to access the survey was advertised in the Italian Journal “Dentista Moderno”. The survey guaranteed in writing that both the results would be anonymized and that a participation consent form would be required to be signed before accessing the questionnaire. The study was conducted in agreement with the ethical standards of the Helsinki’s Declaration.

### Participants

The sample of 162 participants who answered the questionnaire was composed of dentists/orthodontists, hygienists and dental assistants. The requisites for the sample selection were to belong to one of these categories and to wear the FFP2/N95 mask for 4 consecutive hours. Just before the beginning of the workday, oxygen saturation and pulse rate values were recorded without wearing the mask. After four hours of consecutively wearing the FFP2 mask, the pulse oximeter was applied again and the values were recorded with the mask still on. As the questionnaire referred to “normal” working days, the authors of this study decided not to distinguish between those professionals who chose to change the mask after each patient vs. those who kept the masks on for four consecutive hours.

A limitation of this study is the use of different pulse oximeters among 162 participants.

### Study design

This investigation is a cross-sectional observational study which involved a descriptive analysis of a sample of 162 dental health care providers. Data was collected from July to September 2020. The study was designed and executed by the Department of Health, Life and Environmental Science of the University of L’Aquila, Italy. This study received the approval of the Institutional review Board by the Ethics Committee of the University of L’Aquila (Document DR206/2013, 16 July 2013).

### Questionnaire design

A quantitative questionnaire was devised, consisting of seventeen questions (Table 1). The questionnaire was composed of multiple choices, short-answer text, checkboxes and a linear scale. All questions were required to be answered except for one, in which only dentists/orthodontists and/or dental assistants needed to specify the circumstances that required using a N95 mask. A pre-pilot quantitative questionnaire was distributed to 6 colleague dentists aged between 26 and 60 years old to request an ethical approval and to identify any errors that it may have contained, which were subsequently corrected. The authors to this regard found no need to perform a pilot study. The quantitative questionnaire was shared on Google Drive among a sample of 147 Italian and American professionals, with an age ranging from 22 to over 77 years. Thanks to Google Drive it was possible to obtain figures with percentages right away.

**Table 1:**
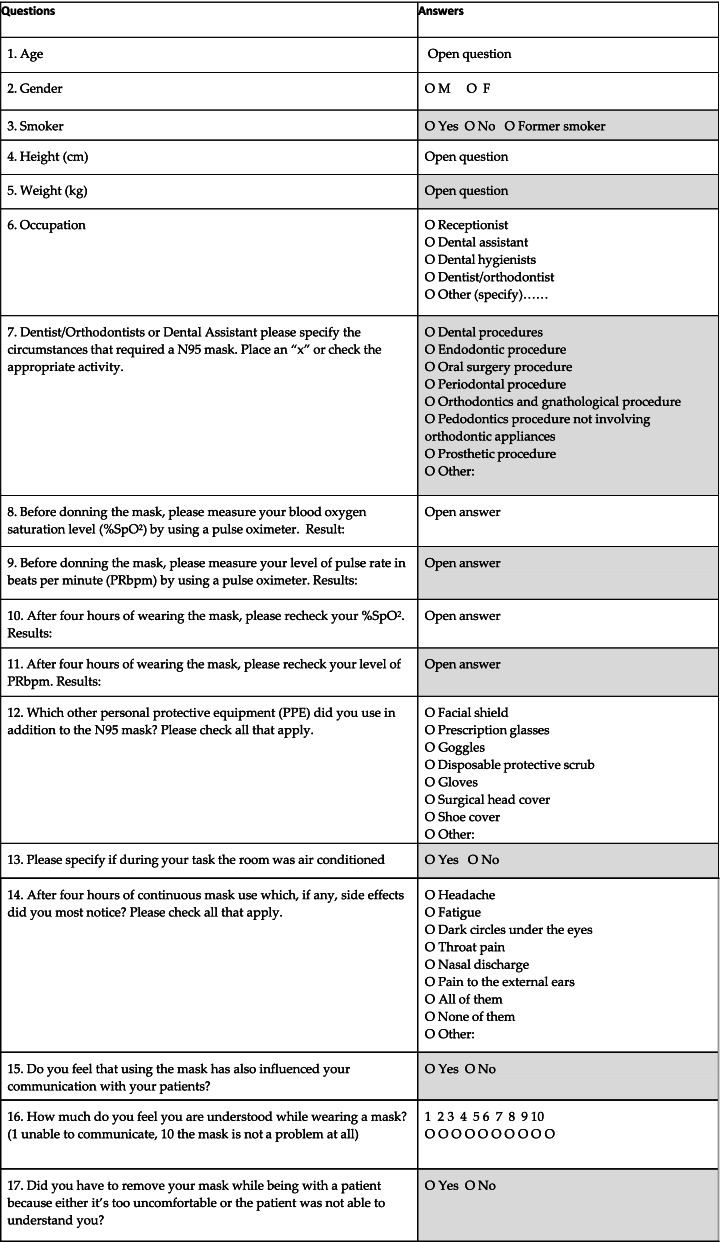
Questionnaire design composed by seventeen questions (included first and last name)

### Statistical analysis

Means and standard deviations were calculated to illustrate continuous variables and data was expressed as a means ± standard deviation (SD). Participants were asked about their weight and height to facilitate the calculation of BMI, which was used to test the validity of the questionnaire. Saturation data was tested for normality using the Kolgomorov-Smirnov test and then the Student T test for paired samples was used to assess a saturation decrease. The Student T test for independent samples was used to assess differences in decrease in subgroups. One Way Anova was used for testing more than two categories and Spearman Rho was used to correlate professional category and binary variables. We performed multinomial logistic regression on factors contributing to the need of mask removal. The software used for the calculation was Statistical Package for the Social Sciences (SPSS 25.0, SPSS Inc., Chicago, USA).

## Results

### Study population

Of the initial 162 participants who responded to the questionnaire 15 were excluded because they stated belonging to a profession other than dentist, hygienists and assistants, or the responses were incomplete.

#### Questions 1, 2–4, 5

A sample of 147 people from Italy and the USA, subjected to a quantitative online questionnaire was considered for final analysis. Among it, the ratio of males to females was 1:1.3, with an average age of 42.9+/-12.0. Demographic information was summarized in Table 2.

**Table 2:** Demographic information of the participants included in the study (*N* = 147)

Variables	n
*Gender*	
Males	62
Females	85
*Age (years) (mean* ± *SD) 42.9* + *12.0*	
Males	45.5 + 13.2
Females	40.9 + 10.8
*Body mass index—BMI (mean* ± *SD) 22.7* ± *3.4*	
Males	24.5 ± 3.0
Females	21.3 ± 3.1

#### Question 3

According to the questionnaire’s responses 25 were smokers and 9 former smokers. Smokers showed a significantly lower baseline SPO^2^ level (97.9±1.0% vs. 98.3±1.2%) and a lower SPO^2^ decrease (0.5±1.0% vs. 1.4±3.0%) while SPO^2^ levels after 4 hours showed no significant differences (97.4±1. vs. 96.9±3.1)

#### Question 6

Regarding the occupation, 101 were dentists/orthodontists, 6 hygienists and 40 dental assistants.

#### Question 7

Regarding the most frequent activities listed in the questionnaire while donning a FFP2, 99 subjects (69%) reported performing conservative (dental) procedures, 58(40%) endodontic procedures, 64(45%) oral surgery procedures, 43(30%) periodontal procedures, 59(41%) orthodontic procedures, and 47(33%) pedodontics procedure not involving orthodontic appliances.

#### Questions 8 and 10

For the entire sample population the baseline saturation was 98.6±1.2 and after four hours of mask wearing there was a significant decrease in oxygen saturation to 97.0±2.9 (*p*<0.01), while there was no significant difference in SPO^2^ decrease between people younger than 50 and those older (1.3±2.0% vs. 1.1±3.2%), or between dentists, assistants or hygienist, and no significant differences were found between degrees of stressful procedures and oxygen desaturation. Facial shields, repellent aprons and air conditioning showed no impact in %SpO^2^ decrease.

#### Questions 9 and 11

Baseline heart rates showed a significant difference between professional groups (Anova *P*<0.05) in particular, the post hoc analysis pointed out a difference between dentists and assistants in which assistants showed an average of 84.0±15.5 bpm vs. 76.05±12.6 bpm in dentists. However, this information needs to be placed in the context of significant gender and age differences that make this aspect *per se* not significant. Moreover, no significant differences were found in the other parameters and heart rate at 4 hours. We assessed potential markers for heart rate differences which could be due to a higher prevalence of female hygienists than in the other categories of the population sample (Spearman’s Rho *p*< 0.01) while age was not significantly different among categories. There were no significant differences between heart rate and SPO^2^ at baseline and after 4 hours and between those who removed the mask often, seldom or never. The combination of masks and other personal protection equipment (PPE), in particular protective screens and scrubs, showed to have no statistically significant influence neither when worn together nor just either one, in both heart rate and SpO^2^ saturation.

#### Questions 12 and 13

Regarding additional personal protective equipment, 115 (78%) responders reported using face shields, 21 (14%) goggles, 141 (96%) protective gloves, 68 (46%) prescription glasses, 104 (71%) protective scrub, 117 (80%) surgical cap, and 26 (25%) shoe cover. The presence of air conditioning while working was mentioned by 99 (60%) subjects (Table 3).

**Table 3:** Use of additional personal protective equipment

***PPE use***	*Face shields*	*Goggles*	*Protective gloves*	*Prescription glasses*	*Protective scrub*	*Surgical cap*	*Shoe cover*	*Use of AC*
***% of use***	*78%*	*14%*	*96%*	*46%*	*71%*	*80%*	*25%*	*99%*

*PPE* Personal protective equipment*, AC Air conditioning*

#### Question 14

Regarding symptoms after 4 hours, the participants reported a median of 2 symptoms, while only 22 (15%) participants reporting no symptoms. The most frequent reported complaints were: 94 (64%) fatigue, 53 (36%) headache, 46 (31%) external ear pain, 25 (17%) dark circles under the eyes (venous pooling), 15 (10%) nasal discharge, and 16 (10%) sore throats (Table 4).

**Table 4:** Symptomatology after 4 h of wearing FFP2 protection masks

***Symptoms after 4hs***	*Fatigue*	*Headache*	*External ear pain*	*Dark circles under eyes*	*Nasal discharge*	*Sore throat*	*No symptoms*
*%*	*64%*	*36%*	*31%*	*17%*	*10%*	*10%*	*15%*

#### Question 15

105 (64%) of the subjects reported that using the mask influenced their communication with their patients. This result though is confounding because 78% of the subjects used both N95 masks and facial shields.

#### Questions 16 and 17

On a non-parametric scale of 1–10 (1 unable to communicate, 10 the mask is not a problem at all) the median for communication score was respectively 7 for removing the mask often, 6 for seldom removing, and 5 for never removing the mask during the 4 h. Participants who removed their masks more often also reported lower communication scores (*P* < 0.001). This aspect alone suggests the necessity of the professional to use augmentative communication devices to facilitate communication among staff and between professionals and patients. In order to assess potential confounding factors, we performed multinomial logistic regression for mask removal frequency using the two significant factors: communication difficulties (question 15) and reporting of headaches (question 14). This regression showed a significant effect only for communication difficulties and thus revealing only a minimal impact of a headache on mask removal. There were no significant correlations between protection devices used and need for mask removal, which was more frequent in those who also reported headaches (*p* < 0.01). There was no statistical difference in total symptoms reported between those who remove masks more often and those who did not. Seventy-five subjects (51%) reported that they had to remove their mask while being with a patient because either it was too uncomfortable or the patient was not able to understand them. When compared with those who had to remove the mask to be understood by patients and the non-parametric scale on question 16, 72 participants stated they never needed to remove the mask (49%), 56 reported seldom (38%) and 19 often (13%), thus there were no significant correlations between protective devices used. Even though the clinician may feel discomfort when using N95 masks with ear bands, it is strongly recommended to avoid removing the mask when still in the working area to avoid risks of contamination with infectious agents that may still be present in the air of the room.

## Discussion

During the COVID-19 pandemic it became apparent how useful protective masks were among health workers and for the whole population [7, 8]. However, the use of the masks for many hours causes problems such as hypercapnia, headaches, shortness of breath, runny nose, dark circles under the eyes, skin lesions, ear pain, or localized compression pain. External ear pain (31%) is one of the most frequent complaints regarding the use of FFP2 masks. This frequent complaint can be avoided by choosing FFP2 masks that have the elastic bands, to properly seal the mask to the face, attached to the head instead of the ones that have these bands attached to the ear. This could be a solution to the referred complaint to eliminate the problem and continue to properly protect the clinician. Another important aspect is the tightness in which the masks are worn because some operators wear masks more loosely than others [9].

Evidence from Smith et al. demonstrated that although N95 respirators appeared to have a protective advantage over surgical masks in laboratory settings, their meta-analysis showed that there was insufficient data to definitively determine whether N95 respirators are superior to surgical masks in protecting health care workers against transmissible acute respiratory infections in clinical settings. [2] According to Fikenzer et al., in healthy individuals, ventilation, cardiopulmonary exercise capacity and comfort are reduced by surgical masks and highly impaired by FFP2/N95 face masks thus significantly impairing the quality of life of their wearer [10].

According to the article published by Nwosu et al, N95 masks have a worse discomfort level than surgical masks but neither masks impacted on the arterial oxygen saturation of the healthcare workers [11].

One longitudinal and prospective observational study performed pre and postoperatively pulse oximeter analysis on 53 surgeons wearing surgical masks. Their findings indicated that the pulse rate of the surgeons increased and SpO^2^ decreased after the first hour. This early change in SpO^2^ may either be due to the facial mask or to the operational stress [3]. To avoid misunderstandings the decrease in SpO^2^ during the first hour is not only related with the use of N95 masks but with the prolonged use of any type of mask.

The results from this study were useful to the speech-language pathologist who collaborated at this project for drafting a list of recommendations for dental professionals that can be useful in increasing communication efficiency between staff and clients and among staff as well. These strategies are also useful in decreasing damage to the overused/dry vocal folds and improving oxygen utilization [12]. For instance, inhaling for four seconds and exhaling for six equates to a breathing rate of six breaths per minute, maximizes many important physiological functions, as well as activating the relaxation centers of the nervous system [13]. It is crucial to reassure professionals who wear a mask for many consecutive hours that it is not a hazard to the general health [14]. A list of strategies to improve wellbeing and communication while wearing a mask is presented in Table 5.

**Table 5:** Strategies to decrease vocal fold damage and discomfort and increase communication efficiency and personal wellbeing

	***Strategies to decrease vocal fold damage and discomfort and increase communication efficiency and personal wellbeing***
***A***	*Breathe through your nose as the nose optimizes air quality, reduces vocal fold damage, facilitates production of nitric oxide (NO) and maximizes oxygen utilization (Sivasankar & Fisher, 2003).*
***B***	*Just before putting on your mask, take five “quality” breaths. With each breath, inhale through the nose for four seconds, exhale through the mouth for six seconds, then rest for two seconds. Repeat these five breaths as soon as you put on the mask, and again after you remove it (McKeowen, 2021)*
***C***	*Keep hydrated as much as possible. The vocal folds are very sensitive to dehydration (Titze, 1988; Verdolini, Titze & Druker, 1990; Chang & Karnell 2004; Sivasankar & Leydon, 2010) and respond with cough and mucus production. When feasible, use a straw to avoid removing the mask completely. When using bottle water to drink, always remember to keep them at a safe distance from any risk potential areas, to avoid contamination of the bottle itself. Always remember to be in a safe area, far from potential risk when drinking water.*
***D***	*Relax your jaw and open your mouth more when talking to avoid muscle tension that transmits to the vocal folds by way of the neck muscles.*
***E***	*Laminate a card with common expressions, allowing the patient to point to the option and/or a card with common procedures that need to be done during the session and that may have not being scheduled.*
***F***	*Use gestures, tone of voice, and body position to augment communication.*
***G***	*Reserve lengthier communications for the end of the procedure when physical distance is possible and the PPE, such as number of masks worn or the combination masks and shield, can be reduced safely.*

As stated in Scarano et al. study, while studying the difference in O2 saturation before and after a surgical procedure, wearing an FFP2 covered by a surgical mask induces a reduction in circulating O_2_ concentrations without clinical relevance, while an increase of heart frequency and a sensation of shortness of breath, light-headedness/headaches were recorded [15].

Although this study provided some useful indications about the impact of wearing FFP2 masks for at least 4 h, there were limitations to the study as well. Some limitations are the reliance on the subjects to document and report the level of SpO^2^, the inability to monitor the continuous use of the mask or the correct use of the mask, the arbitrary decision to test after 4 h without intermediate tests and the lopsided sample in which the category of dentists/orthodontists vastly outnumbered that of dental hygienists, even if both work in close proximity to the mouth of the patient. Another limitation is related to the fact that according to the results, the use of N95 masks could seem to be responsible for impaired communication with the patient. The majority of the subjects (78%) though used facial shields together with N95 masks, thus this result could be mistaken because the use of a facial shield strongly influences the communication with the patient.

The experience gathered from this study indicates the need, in the future, to enroll a professional psychologist to refine the questionnaire, the inclusive and exclusive criteria and to better control any possible biases embedded in the questions.

## Conclusions

The purpose of this cross-sectional observational study was to evaluate the effects of SpO^2^ in a sample of dental health care providers who wear a N95 mask (FFP2) for four consecutive hours, measured by a pulse oximeter before donning the mask and again after four hours of work. The results confirmed what the dental professionals, have known anecdotally that the use of the FFP2 masks, although certainly representing a safeguard for the health care worker, also presents some side effects, such as headaches, fatigue and external earache, which may affect the professional’s quality of life during working hours. The study highlights a significant decrease in oxygen saturation after only 4 hours of work, but it also highlighted how mask wearing impaired communication with patients. This aspect and the need for better communications can lead the operators to remove the mask to improve breathing and communication, thus putting themselves at risk of infection. Of all the aspects explored in this study, the most interesting was the impact on fatigue and communication because some strategies can be proposed to improve this situation. The strategies proposed in this article can easily be implemented to reduce headaches and fatigue by improve breathing efficiency and to improve communication while donning a mask by improving voice quality and using augmentative tools.

## Data Availability

The data that support the findings of this study are available from the corresponding author (S.S.) upon reasonable request.

## References

[1] Bein B, Bachmann M, Huggett S, Wegermann P (2020). SARS-CoV-2/COVID-19: Empfehlungen zu Diagnostik und Therapie [SARS CoV-2/COVID-19: evidence-based recommendation on diagnosis and therapy]. Anasthesiol Intensivmed Notfallmed Schmerzther.

[2] Li KKW, Joussen AM, Kwan JKC, Steel DHW (2020). FFP3, FFP2, N95, surgical masks and respirators: what should we be wearing for ophthalmic surgery in the COVID-19 pandemic?. Graefes Arch Clin Exp Ophthalmol.

[3] Beder A, Büyükkoçak U, Sabuncuoğlu H, Keskil ZA, Keskil S (2008). Preliminary report on surgical mask induced deoxygenation during major surgery. Neurocirugia (Astur).

[4] Jubran A (2015). Pulse oximetry. Crit Care..

[5] Abdulla J, Laursen LC, Thomsen CB (1999). Arteriepunktur eller pulsoksimetri? [Arterial puncture or pulse oximetry?]. Ugeskr Laeger.

[6] Bowes WA, Corke BC, Hulka J (1989). Pulse oximetry: a review of the theory, accuracy, and clinical applications. Obstet Gynecol.

[7] Fikenzer S, Uhe T, Lavall D, et al. Effects of surgical and FFP2/N95 face masks on cardiopulmonary exercise capacity [published online ahead of print, 2020 Jul 6]. Clin Res Cardiol. 2020;1–9. 10.1007/s00392-020-01704-y. 10.1007/s00392-020-01704-y PMC7338098 32632523

[8] Boyd K, Saccomanno S, Lewis CJ, Coceani Paskay L, Quinzi V, Marzo G (2021). Myofunctional therapy. Part 1: Culture, industrialisation and the shrinking human face. Eur J Paediatr Dent.

[9] Powell JB, Kim JH, Roberge RJ. Powered air-purifying respirator use in healthcare: effects on thermal sensations and comfort. J Occup Environ Hyg. 2017;14(12):947–54. 10.1080/15459624.2017.1358817. 10.1080/15459624.2017.1358817 PMC6198805 28763290

[10] McKeown P (2021). The Breathing Cure. Pag 170 and 756.

[11] Nwosu ADG, Ossai EN, Onwuasoigwe O, Ahaotu F. Oxygen saturation and perceived discomfort with face mask types, in the era of COVID-19: a hospital-based cross-sectional study. Pan Afr Med J. 2021;16(39):203. 10.11604/pamj.2021.39.203.28266. 10.11604/pamj.2021.39.203.28266 PMC8464215 34603584

[12] U.S. Centers for Disease Control and Prevention, "Use of cloth face coverings to help slow the spread of COVID-19," May 23, 2020. http://www.cdc.gov/coronavirus/2019-ncov/prevent-getting-sick/diy-cloth-face-coverings.html.

[13] Quinzi V., Coceani Paskay L., Manenti R. J., Giancaspro S., Marzo G., Saccomanno S. Telemedicine for a multidisciplinary assessment of orofacial pain in a patient affected by Eagle’s Syndrome: a clinical case report. Open Dent. J. 2021. 10.2174/1874210602115010102.

[14] Saccomanno S, Quinzi V, Sarhan S, Lagana’ D, Marzo G (2020). Perspectives of tele-orthodontics in the COVID 19 emergency and as a future tool in daily practice. Eur J Paediatric Dent.

[15] Scarano A, Inchingolo F, Rapone B, Festa F, Rexhep Tari S, Lorusso F (2021). Protective Face Masks: Effect on the Oxygenation and Heart Rate Status of Oral Surgeons during Surgery. Int J Environ Res Public Health.

